# An unusual presentation of a Gastrointestinal stromal tumour (GIST)

**DOI:** 10.1186/1477-7819-5-78

**Published:** 2007-07-14

**Authors:** Solomon KP John, Sanjoy Basu, Richard J Lawrance, Nick Davies

**Affiliations:** 1 Department of Surgery, Royal Bournemouth Hospital, Bournemouth, UK

## Abstract

**Background:**

Gastrointestinal stromal tumours (GIST) are rare tumours, now more frequently identified with the new imaging modalities like computerised tomography (CT) and magnetic resonance imaging (MRI). We report a rare presentation of a GIST with an unusual diagnostic workup in a multidisciplinary setting leading to a definitive diagnosis and treatment.

**Case presentation:**

A 55-year-old lady was admitted under the general surgeons, with 3-day history of abdominal pain, three-week history of loss of appetite and weight. The patient was sequentially investigated with ultrasonography, computerised tomography and finally selective angiogram in a multidisciplinary setting. The selective angiogram showed a GIST with intratumour bleed, leading to successful surgical excision and being recurrence free at 22 month follow up.

**Conclusion:**

Clinical presentation of these tumours can be varied and gastrointestinal bleeding is the commonest mode described in the literature. The clinician needs to be aware of much more rare presentations of the GIST including an intra tumour bleed. A structured multidisciplinary approach would lead to successful diagnosis and treatment.

## Background

Gastrointestinal stromal tumours (GISTs) are rare, representing 0.1–3% of all gastrointestinal cancers with an estimated incidence of 15 per million [1-3]. GISTs, previously thought to arise from the interstitial cells of Cajal [4], are presently believed to originate from the common intestinal mesenchymal precursor cell [5,6] and are characterised by the over-expression of the tyrosin kinase receptor KIT. The stomach is the most common site for GISTs (60%), with 15% in the small and large bowel [7]. Presentation depends on its size and location [8]. Gastrointestinal tract bleeding (50%) is the most common presentation, followed by abdominal pain (20–50%), obstruction (20%) and approximately one third are detected incidentally [1]. We report a case of GIST arising in the stomach presenting with an 'intra-tumour bleed'.

## Case presentation

A 55-year-old woman was admitted under the general surgeons with a three-day history of colicky right hypochondrial and epigastric pain together with three-weeks of reduced appetite and weight loss of 5 kilograms. Abdominal examination revealed mild tenderness in the right upper quadrant. Routine investigation revealed haemoglobin of 11.6 gm/dl and marginally elevated alkaline phosphatase of 110 mg/dl. Abdominal ultrasound scans revealed a 7.2 × 6.8 cm thin-walled partially cystic and solid structure adjacent to the inferior margin of the left lobe of liver.

**Figure 1:**
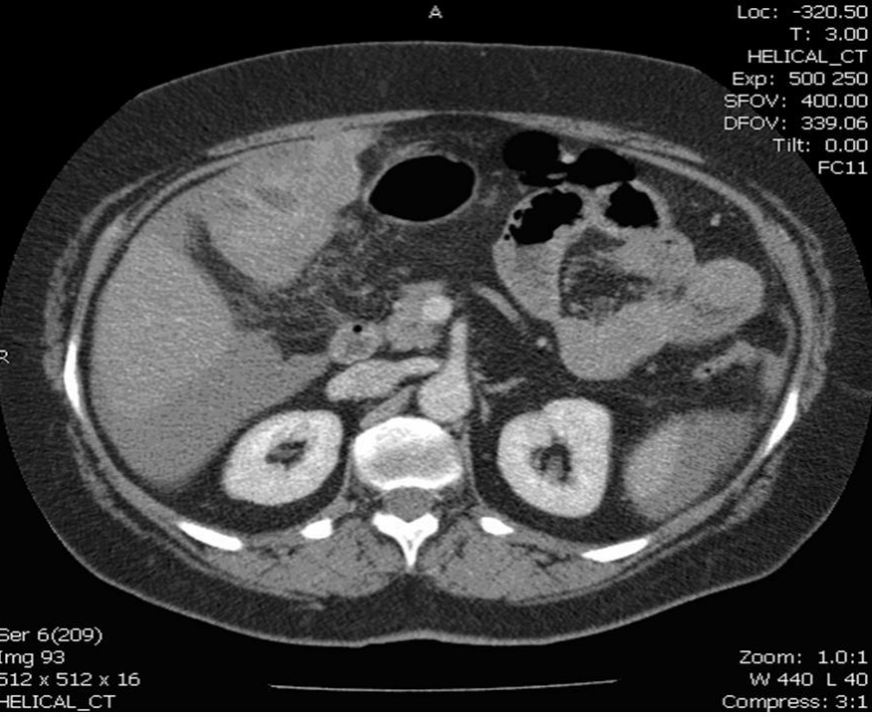
CT scan picture of the GIST close to liver and distinctly separate from gastric lumen.

**Figure 2:**
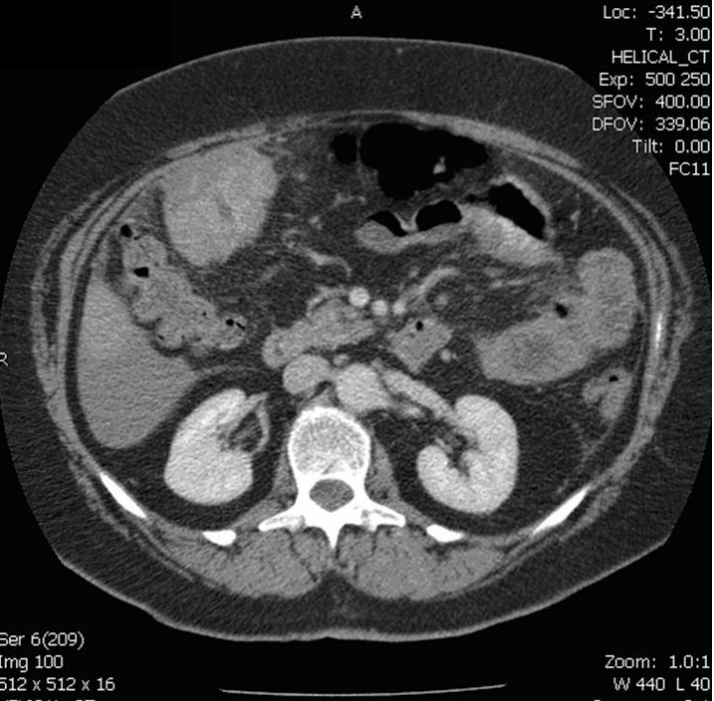
CT scan picture of enhancing lower extent of GIST visualized in relation to hepatic flexure of colon.

Computerised tomography (CT) of the abdomen confirmed a partly cystic and partly solid mass with some contrast enhancement and low volume ascitis (figure 1, 2). She was discussed in the upper gastrointestinal multidisciplinary team meeting. The mass was thought to be a bleeding liver lesion. Selective mesenteric and hepatic angiography with a view to embolisation was performed.

**Figure 3:**
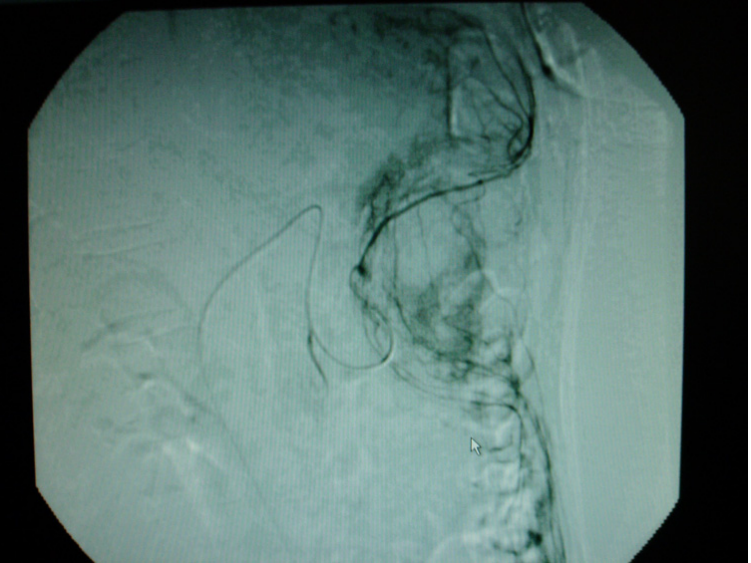
Tumour neo-vascularisation on distal gastro duodenal and gastro-epiploic catheterisation during angiogram (White arrow as pointer).

Angiography showed normal hepatic artery architecture. Distal gastroduodenal and gastro-epiploic catheterisation exhibited tumour neo-vascularisation (figure 3), which was not amenable to embolisation. The appearance was thought to be consistent with either a GIST or a liposarcoma due to the hypervascular picture with many early filling abnormal vessels, unusually sustained tumour blush and prominent draining veins. At laparotomy there was an exophytic lesion arising from the antrum of the stomach with signs of bleeding into the tumour. The tumour was locally excised with clear margins.

The histology confirmed a 4.5 × 3.5 cm GIST with evidence of recent haemorrhage, strongly positive for CD117 with negative resection margins and a favourable mitotic number of <5/50 HPF. The patient made an uneventful recovery and has remained recurrence free for 22 months.

## Discussion

GISTs can present in a number of different ways and are often diagnosed incidentally. In a population-based study, approximately 70% patients with GIST were symptomatic, 20% asymptomatic and 10% detected at autopsy. GISTs causing symptoms tended to be large with a mean size of 6 cm as opposed to 2 cm asymptomatic GISTs and 1.5 cm GISTs detected at autopsy [2].

Symptoms caused by GISTs are commonly due to their location with both mass effect and intraluminal bleeding being reported. Large GISTs can cause vague abdominal discomfort, pain, bloating, early satiety or increased abdominal girth. Erosion into the gastrointestinal tract can induce significant hemorrhage causing haematemesis, malena or anaemia from occult bleeding. They have also been noted to cause dysphagia in the oesophagus, biliary obstruction around the ampulla of Vater, intussusception or intestinal obstruction in the small bowel. Other rare presentations described in the literature include hypoglycemia [9], abdominal pain due to torsion of an exophytic tumour, presentation as a content in a hernial sac, intraperitoneal bleed [10,11] and mimicking acute appendicitis [12].

In this case increased CT enhancement and close proximity to the liver provided a differential diagnosis of a haemangioma, liver cyst or a vascular metastasis. Further vascular investigations were required to make the diagnosis. An extensive literature review has not shown any report of a GIST presenting with an 'intratumour bleed'. This case demonstrates another unusual presentations for this rare mesenchymal tumour.

## Conclusion

Clinical presentation of these tumours can be varied and gastrointestinal bleeding is the commonest mode described in the literature. The clinician needs to be aware of much more rare presentations of the GIST including an intra tumour bleed. A structured multidisciplinary approach would lead to successful diagnosis and optimal treatment for the patient in these unusual clinical situations.

## Competing interests

The author(s) declare that they have no competing interests.

## Authors' contributions

SKPJ: Conceived the idea, carried out literature review, wrote the manuscript.

SB: Contributed by carrying out literature review and drafting the manuscript.

RJL: Overall supervision of manuscript preparation and proof reading.

ND: Overall supervision of manuscript preparation and proof reading.

All authors read and approved the final manuscript.

## References

[B1] Rossi CR, Mocellin S, Mancarelli R, Foletto M, Pilati P, Nitti D, Lise M (2003). Gastrointestinal stromal tumours: from a surgical to a molecular approach. Int J Cancer.

[B2] Kindblom LG (2003). Education Session E450, oral presentation "Gastrointestinal Stromal Tumours Diagnosis, Epidemiology and Prognosis" in Gastrointestinal Stromal Tumour:Current management and Future Challenges. Chair: Blanke CD ASCO.

[B3] Corless CL, Fletcher JA, Heinrich MC (2004). Biology of gastrointestinal stromal tumours. J Clin Oncol.

[B4] Kindblom LG, Remotti HE, Aldenborg F, Meis-Kindblom JM (1998). Gastrointestinal stromal tumours show phenotypic characteristics of the interstitial cells of Cajal. Am J Pathol.

[B5] De Silva MVC, Reid R (2003). Gastrointestinal Stromal Tumours (GIST): C-kit Mutations, CD 117 Expression, Differtial Diagnosis and Targeted Cancer Therapy with Imatinib. Pathol Oncol Res.

[B6] Wingen CBM, Pauwels PAA, Debies-Rychter M, Van Gemert WG, Vos MC (2005). Uterine gastrointestinal Stromal tumour (GIST). Gynaecol Oncol.

[B7] Miettinen M, Virolainen M, Maarit Sarlomo R (2005). Gastrointestinal stromal tumour of the stomach: a clinicopathologic, immunohistochemical and molecular genetic study of 1765 cases with long-term follow-up. Am J Surg Pathol.

[B8] Fletcher CD, Bermenn JJ, Corless C, Gorstein F, Lasota J, Longley BJ, Miettinen M, O'Leary TJ, Remotti H, Rubin BP, Shmookler B, Sobin LH, Weiss SW (2002). Diagnosis of gastrointestinal stromal tumours: a consensus approach. Hum Pathol.

[B9] Beckers MMJ, Slee PHT, van Doorn J (2003). Hypoglycaemia in a patient with a gastrointestinal stromal tumour. [Letter]. Clinical Endocrinology.

[B10] Dubenec SR, Dawes-Higgs EK, Higgs RJED, Truskett PG (2001). Haemoperitoneum caused by spontaneous rupture of a gastrointestinal stromal tumour. ANZ J Surg.

[B11] Cegarra-Navarro MF, de la Calle MA, Girela-Baena E, García-Santos JM, Lloret-Estañ F, de Andrés EP (2005). Ruptured gastrointestinal stromal tumors: radiologic findings in six cases. Abdom Imaging.

[B12] Ajduk M, Mikulic D, Sebecic B, Gasparov S, Patrlj L, Erdelez L (2004). Spontaneously ruptured gastrointestinal stromal tumor (GIST) of the jejunum mimicking acute appendicitis. Coll Antropol.

